# Supplementary material for: Tracing the substrate translocation mechanism in P-glycoprotein
Source: eLife. 2024 Jan 23;12:RP90174. doi: 10.7554/eLife.90174 (PMC10945689; doi:10.7554/eLife.90174)
Supplement: Supplementary file 1. [file elife-90174-supp1.docx]

**Supplementary File 1. Cryo-EM data collection, refinement, and validation statistics.**

|  | OF335-apo  (EMD- 15687)  (PDB 8AVY) | OF335-nolig  (EMD- 14754)  (PDB 7ZK4) | OF335-1lig  (EMD- 14755)  (PDB 7ZK5) | OF335-2lig  (EMD- 14756)  (PDB 7ZK6) | IF335-2lig  (EMD-17630)  (PDB 8PEE) |
| --- | --- | --- | --- | --- | --- |
| **Data collection** |  |  |  |  |  |
| Microscope | Titan Krios G3i | Titan Krios G3i | Titan Krios G3i | Titan Krios G3i | Titan Krios G3i |
| Energy filter and camera  Energy filter slit width | Gatan BioQuantum K3  30 eV | Gatan BioQuantum K3  30 eV | Gatan BioQuantum K3  30 eV | Gatan BioQuantum K3  30 eV | Gatan BioQuantum K3  30 eV |
| Voltage (kV) | 300 | 300 | 300 | 300 | 300 |
| Nominal magnification | 105,000 | 105,000 | 105,000 | 105,000 | 105,000 |
| Pixel size (Å) | 0.837 | 0.837 | 0.837 | 0.837 | 0.837 |
| Electron exposure (e^–^/Å^2^) | 75 | 75 | 75 | 75 | 75 |
| Total exposure time (s) | 4 | 4 | 4 | 4 | 4 |
| Number of frames per image | 50 | 50 | 50 | 50 | 50 |
| Total number of images | 6594 | 18394 | 18394 | 18394 | 13999 |
| Defocus range (μm) | -1.2 to -2.5 | -1 to -2.5 | -1 to -2.5 | -1 to -2.5 | -1 to -2.5 |
| **Image processing** |  |  |  |  |  |
| Processing software | cryoSPARC | cryoSPARC | cryoSPARC | cryoSPARC | RELION 3.1 |
| Motion correction software | cryoSPARC | cryoSPARC | cryoSPARC | cryoSPARC | RELION3.1 |
| CTF estimation software | cryoSPARC | cryoSPARC | cryoSPARC | cryoSPARC | Gctf |
| Particle selection software | Topaz | Topaz | Topaz | Topaz | RELION 3.1 |
| Initial particle images (no.) | 2,262,305 | 9,065,013 | 9,065,013 | 9,065,013 | 2,975,426 |
| Final particle images (no.) | 790,159 | 1,437,110 | 1,184,253 | 939,924 | 218,872 |
| Final refinement software | cryoSPARC | cryoSPARC | cryoSPARC | cryoSPARC | cryoSPARC |
| Symmetry imposed | C1 | C1 | C1 | C1 | C1 |
| Map resolution (Å) | 2.9 | 2.6 | 2.6 | 3.1 | 3.8 |
| B-factor (Å^2^) | 138.2 | 119.3 | 118.6 | 154.8 | 155.2 |
|  |  |  |  |  |  |
| **Refinement statistics** |  |  |  |  |  |
| Initial model (PDB code) | 6C0V | 6C0V | 6C0V | 6C0V | 4Q9I |
| Modeling software | Coot, PHENIX | Coot, PHENIX | Coot, PHENIX | Coot, PHENIX | Coot, PHENIX |
| Model composition  Non-hydrogen atoms  Protein residues  Water molecules  Ligands | 9433  1175  4  12 | 9371  1176  4  9 | 9436  1177  33  10 | 9267  1177  4  7 | 9217  1157  -  7 |
| Mean *B* factors (Å^2^)  Protein  Water molecules  Ligand | 27.15  -  37.03 | 27  -  59 | 39  37  44 | 59  30  50 | 71  -  57 |
| R.m.s. deviations  Bond lengths (Å)  Bond angles (°) | 0.030  1.265 | 0.024  1.04 | 0.024  1.16 | 0.019  0.91 | 0.024  1.040 |
| Validation  MolProbity score  Clashscore  Poor rotamers (%) | 1.50  9.39  0.74 | 1.50  9.29  0.10 | 1.56  9.43  0.10 | 1.49  9.33  0.10 | 2.00  12.52  0.32 |
| Ramachandran plot  Favored (%)  Allowed (%)  Disallowed (%) | 98.04  1.96  0.00 | 97.95  2.05  0.00 | 97.70  2.22  0.08 | 98.12  1.79  0.09 | 94.19  5.81  0.00 |

|  | OF971-1lig (EMD- 14758)  (PDB 7ZK8) | IF971-1lig  (EMD- 14759)  (PDB 7ZK9) | OF978-1lig  (EMD- 14760)  (PDB 7ZKA) | IF978-2lig  (EMD- 14761)  (PDB 7ZKB) |
| --- | --- | --- | --- | --- |
| **Data collection** |  |  |  |  |
| Microscope | Titan Krios G4 | Titan Krios G3i | Titan Krios G4 | Titan Krios G3i |
| Energy filter and camera  Energy filter slit width | Selectris X Falcon 4  10 eV | Gatan BioQuantum K3  30 eV | Selectris X Falcon 4  10 eV | Gatan BioQuantum K3  30 eV |
| Voltage (kV) | 300 | 300 | 300 | 300 |
| Nominal magnification | 215,000 | 105,000 | 215,000 | 105,000 |
| Pixel size (Å) | 0.537 | 0.837 | 0.537 | 0.837 |
| Electron exposure (e^–^/Å^2^) | 70 | 75 | 70 | 75 |
| Total exposure time (s) | 4 | 4 | 4 | 4 |
| Number of frames per image | 70 | 50 | 70 | 50 |
| Total number of images | 12128 | 6758 | 14998 | 6204 |
| Defocus range (μm) | -1 to -2.5 | -1 to -2.5 | -1 to -2.5 | -1 to -2.5 |
| **Image processing** |  |  |  |  |
| Processing software | cryoSPARC | cryoSPARC | cryoSPARC | cryoSPARC |
| Motion correction software | cryoSPARC | cryoSPARC | cryoSPARC | cryoSPARC |
| CTF estimation software | cryoSPARC | cryoSPARC | cryoSPARC | cryoSPARC |
| Particle selection software | Topaz | cryoSPARC | cryoSPARC | cryoSPARC |
| Initial particle images (no.) | 1,644,112 | 1,664,538 | 1,761,489 | 1,744,204 |
| Final particle images (no.) | 298,192 | 443,812 | 271,467 | 343,873 |
| Final refinement software | CryoSPARC | CryoSPARC | CryoSPARC | CryoSPARC |
| Symmetry imposed | C1 | C1 | C1 | C1 |
| Map resolution (Å) | 3.0 | 4.3 | 2.9 | 4.7 |
| B-factor (Å^2^) | 128.1 | 285 | 116.8 | 347.4 |
|  |  |  |  |  |
| **Refinement statistics** |  |  |  |  |
| Initial model (PDB code) | 6C0V | 4Q9I | 6C0V | 4Q9I |
| Modeling software | Coot, PHENIX | Coot, PHENIX | Coot, PHENIX | Coot, PHENIX |
| Model composition  Non-hydrogen atoms  Protein residues  Water molecules  Ligands | 9331  1181  4  7 | 9004  1157  -  1 | 9407  1177  4  9 | 9035  1157  -  2 |
| Mean *B* factors (Å^2^)  Protein  Water molecules  Ligand | 56  33  55 | 181  -  154 | 31  12  37 | 121  -  71 |
| R.m.s. deviations  Bond lengths (Å)  Bond angles (°) | 0.016  1.00 | 0.005  0.92 | 0.003  0.59 | 0.004  0.80 |
| Validation  MolProbity score  Clashscore  Poor rotamers (%) | 1.74  9.69  0.10 | 2.07  15.76  0.21 | 1.69  9.86  0.00 | 2.08  13.26  0.00 |
| Ramachandran plot  Favored (%)  Allowed (%)  Disallowed (%) | 96.52  3.48  0.00 | 94.62  5.29  0.09 | 97.02  2.90  0.08 | 93.06  6.94  0.00 |
