# Supplementary material for: Tracing the substrate translocation mechanism in P-glycoprotein
Source: eLife. 2024 Jan 23;12:RP90174. doi: 10.7554/eLife.90174 (PMC10945689; doi:10.7554/eLife.90174)
Supplement: Supplementary file 2. [file elife-90174-supp2.docx]

**Supplementary File 2. Summary of cryo-EM data for the Pgp mutants.**

| **Pgp mutants** | **Structures** | **Resolution** | **Conformation** | **Bound cyclopeptides** | **Bound nucleotides** |
| --- | --- | --- | --- | --- | --- |
| Pgp335 (L335C/TM6) | IF335-2lig | 3.8 Å | Inward-facing | 2 ligands (1 covalent and 1 non-covalent) | Not present |
|  | OF335-apo | 2.9 Å | Outward-facing | No ligand | ATP |
|  | OF335-nolig | 2.6 Å | Outward-facing | No ligand | ATP |
|  | OF335-1lig | 2.6 Å | Outward-facing | 1 ligand (covalent) | ATP |
|  | OF335-2lig | 3.1 Å | Outward-facing | 2 ligands (1 covalent and 1 non-covalent) | ATP |
| Pgp978 (V978C/TM12) | IF978-2lig | 4.7 Å | Inward-facing | 2 ligands (1 covalent and 1 non-covalent | Not present |
|  | OF978-1lig | 3.0 Å | Outward-facing | 1 ligand (covalent) | ATP |
| Pgp971 (L971C/TM12) | IF971-1lig | 4.3 Å | Inward-facing | 1 ligand (non-covalent) | Not present |
|  | OF971-1lig | 3.0 Å | Inward-facing | 1 ligand (covalent) | ADP/vanadate |
