# Supplementary material for: Tracing the substrate translocation mechanism in P-glycoprotein
Source: eLife. 2024 Jan 23;12:RP90174. doi: 10.7554/eLife.90174 (PMC10945689; doi:10.7554/eLife.90174)
Supplement: Supplementary file 3. [file elife-90174-supp3.docx]

**Supplementary File 3: Equilibration protocol for MD simulations**.

| Equilibration step | Time  [$\boldsymbol{ps}$] | Timestep $\left[ \boldsymbol{ps} \right]$ | Ensemble | Thermostat | Barostat | Force constant backbone $\left[ \frac{\boldsymbol{kJ}}{\boldsymbol{mol}\boldsymbol{nm}^{\boldsymbol{2}}} \right]$ | Force constant side chain $\left[ \frac{\boldsymbol{kJ}}{\boldsymbol{mol}\boldsymbol{nm}^{\boldsymbol{2}}} \right]$ | Force constant lipids$\left[ \frac{\boldsymbol{kJ}}{\boldsymbol{mol}\boldsymbol{nm}^{\boldsymbol{2}}} \right]$ | Force constant dihedrals$\left[ \frac{\boldsymbol{kJ}}{\boldsymbol{mol}\boldsymbol{rad}^{\boldsymbol{2}}} \right]$ |
| --- | --- | --- | --- | --- | --- | --- | --- | --- | --- |
| 1 | 125 | 0.001 | NVT | Berendsen |  | 4000 | 2000 | 1000 | 1000 |
| 2 | 125 | 0.001 | NVT | Berendsen |  | 2000 | 1000 | 400 | 400 |
| 3 | 125 | 0.001 | NPT | Berendsen | Berendsen (semi-isotropic) | 1000 | 500 | 400 | 200 |
| 4 | 500 | 0.002 | NPT | Berendsen | Berendsen (semi-isotropic) | 500 | 200 | 200 | 200 |
| 5 | 500 | 0.002 | NPT | Berendsen | Berendsen (semi-isotropic) | 200 | 50 | 40 | 100 |
| 6 | 500 | 0.002 | NPT | Berendsen | Berendsen (semi-isotropic) | 50 | 0 | 0 | 0 |
